# Letter from R. D. Addington

**Published:** 1846-06

**Authors:** R D. Addington


### ARTICLE IV.

### *Letter from* R. D. Addington.

Wilmington, N. C., *April* 20*th*, 1846.

Dr. Chapin A. Harris,

Sir:—In accordance with a promise I made last spring, preparatory to my departure for Rio de Janeiro, that I would inform you "What was the present condition of Dental Surgery in Brazil?" I shall, as far as I am able, with the opportunities afforded me of judging correctly, enter into the subject with the intention of giving a candid statement of facts.

During my sojourn in Brazil, I visited about twelve cities and large towns, in most of which I found one or more French or Brazilian dentists, and the greater part of them totally unfit, by their education and qualifications, to render their operations effectual; the materials used are equally so, being lead, amalgams and common French foil, which they called gold; the prices for filling with the two first are 666rs. or about 33⅓ cents, for the latter 1000rs. or 50 cents.

When these prices for operations are compared with our expenses, which, even in villages, is 2000rs.* per day, whether you remain one or three hundred and sixty-five, without the practice equalling by one-eighth that of towns of the same size in the United States, must render an American's prospects gloomy indeed, especially when he has to contend with the above dentists, who live with their Brazilian wives, who perform the duties of servants for them, on the poorest fare, in secluded hovels. They are accustomed to live merely sustaining nature, and if their business will afford them a livelihood, they are contented. You must not suppose I am confining my remarks to villages, for they are much worse, but towns numbering from ten to twenty thousand inhabitants.

I can better illustrate the practice of these places, by mentioning one of several cases which came under my observation. Immediatety on my arrival at the seaport (Santos) of the Province of St. Paul, a town of ten thousand inhabitants, Mr. Geo. Black, the American consul, to whom I carried letters of introduction, called and informed me that the British consul and several others had heard of my arrival, and wished operations performed in their families; as I was still debilitated by my long voyage from the United States, I declined commencing my business until I was sufficiently recruited, but accepted his invitation to extend my acquaintance. We called on the consul, who Mr. Black previously informed me was the wealthiest and most intelligent gentleman in the place; I found him polite and affable, but when I informed him my prices were the same as Mr. Louis Burdell's of Rio, he seemed much astonished at the assumption, mentioned that the prices of a Brazilian who re-

*2000rs.=$1 00.

sided in the town were about one-eighth of that amount, that the people out of the capital were poor, &c., and if I consented to work at his prices, he *might* find something for me to do; although I informed him my materials were nearly worth that amount, it produced no better effect than to decline my services, to which the rest followed suite. I afterwards called upon this dentist, who appeared delighted with my artificial teeth, but would not offer me more than half they cost, he being governed in their value by some worthless French teeth he showed me. My foils met the same fate. This man showed me, at my request, his instruments, &c., which he kept in a small soap box; with difficulty I kept my countenance, and proceeded to ascertain the uses to which he applied them, &c. This is the dentist of Santos, who confessed he had read nothing on the subject—had conversed with two French dentists, and spent about two hours in the office of one of them, during a visit to Rio.

Although these towns may be surrounded by the wealthy sugar and coffee plantations, you need not expect patronage from their owners, as they either live on them very secluded, or the refined and intelligent portion live in the large cities.

I deem it necessary, to be better understood, to mention that very little refinement and social intercourse exist out of the cities of Rio, Bahia and Pernambuco, and in them confined mostly to the men.

From dentistry in the towns, I shall endeavor to show what it is in the three largest cities of the empire. Two gentlemen from New York settled in Pernambuco about 1842; from thence they went to Rio, where they remained about twelve months, and removed to Buenos Ayres, thence to Lisbon, thence to Gibraltar, thence to Bahia, where they dissolved copartnership, one remaining in that city, and the other returned to Pernambuco; they cannot be very successful, from what I could learn and the *subjects* of letters from them shown to me at Rio.

Within the last fifteen years, about twenty American dentists have opened offices in the capital, of whom I know nothing; but two of my countrymen are now practicing in the city; one of them early prepossessed me against the supposed course of practice pursued in that country, from his mentioning his own, backed by the assertion, that although he knew it was incorrect, he had to adopt it from the *impatience* of his patients; however mortifying this might be to me, it was destined to be of short duration, as I, the following day, had a long conversation with Mr. Louis Burdell, and I left his office highly gratified that he, at least, of all in the country, practiced what are now considered, in the United States, the correct principles of dental surgery.

This gentleman has been in the country nearly six years, during which he has had many difficulties to contend with; but since his services were required at the palace, his business has become enormous, requiring his and an assistant's undivided attention from 7 A. M. until 4 P. M.; during this time his operating and sitting rooms are filled with the wealthiest and most distinguished families of the empire, where they were seen by me *patiently* waiting for his services, amusing themselves with musical instruments, books, gossip, &c., and if not waited on, they would return day after day, until their wishes could be gratified. Whilst this constant exhibition of their wishes to obtain his services are seen, I have repeatedly visited the offices of the others, and never found more than one, and generally that one a foreigner or plebian, endeavoring to cheapen the prices of their operation. In opposition to this state of affairs with the others, I will mention one of many incidents approving of Mr. L. Burdell, which occurred in my presence. One of the emperor's ministers, whose daughter, about thirteen years of age, had her right lateral incisor so irregular as to come behind the lower teeth, desired it to be placed in the line; which was pronounced finished after she had visited his office about four times. The father desired to know his charge, which Dr. B. valued at 75000rs., to which the minister objected, and requested a charge equal to his means, and proposed 150,000rs., which Dr. B. declined to accept, as the charge was amply sufficient; the minister hesitated a few moments, then seeming to acquiesce, handed Dr. B. a 1,000,000rs. note, which he took and proceeded to get his change, when the minister withdrew, congratulating himself at the success of the deception.

By his success I do not wish wrong conclusions to be drawn, which an American is liable to without knowing the character of that people. When they become satisfied with one's services, they remain constant in their patronage, and unaffected by innovations like our countrymen; this must act greatly to the disadvantage of any one now commencing dentistry in Rio de Janeiro, whatever his qualifications. Again, those who desire and are able to pay for the services of a skilful dentist, are very few when compared to the population of Brazil, and those few centre at Rio, and will not divide their patronage from opposition.

I do not doubt but time, if peace is continued within the provinces of Brazil, which is not altogether certain, will cause changes sufficient to induce the further emigration of our profession to the other cities. Every thing must have ample time with them to effect an object, which induces the opinion that the present state of the country and people should keep away the profession, unless it strikes at Rio de Janeiro.

All of which is respectfully submitted to your consideration, to be used as seems most fit.

Yours, &c. &c.

R D. ADDINGTON.

